# Updating appetitive memory during reconsolidation window: critical role of cue-directed behavior and amygdala central nucleus

**DOI:** 10.3389/fnbeh.2013.00186

**Published:** 2013-12-09

**Authors:** Megan E. Olshavsky, Bryan J. Song, Daniel J. Powell, Carolyn E. Jones, Marie-H. Monfils, Hongjoo J. Lee

**Affiliations:** ^1^Department of Psychology, The University of Texas at AustinAustin, TX, USA; ^2^Center for Learning and Memory, The University of Texas at AustinAustin, TX, USA

**Keywords:** appetitive learning, fear learning, conditioned orienting, extinction, central amygdala

## Abstract

When presented with a light cue followed by food, some rats simply approach the foodcup (Nonorienters), while others first orient to the light in addition to displaying the food-cup approach behavior (Orienters). Cue-directed orienting may reflect enhanced attentional and/or emotional processing of the cue, suggesting divergent natures of cue-information processing in Orienters and Nonorienters. The current studies investigate how differences in cue processing might manifest in appetitive memory retrieval and updating using a paradigm developed to persistently attenuate fear responses (Retrieval-extinction paradigm; Monfils et al., 2009). First, we examined whether the retrieval-extinction paradigm could attenuate appetitive responses in Orienters and Nonorienters. Next, we investigated if the appetitive memory could be updated using reversal learning (fear conditioning) during the reconsolidation window (as opposed to repeated unreinforced trials, i.e., extinction). Both extinction and new fear learning given within the reconsolidation window were effective at persistently updating the initial appetitive memory in the Orienters, but not the Nonorienters. Since conditioned orienting is mediated by the amygdala central nucleus (CeA), our final experiment examined the CeA’s role in the retrieval-extinction process. Bilateral CeA lesions interfered with the retrieval-extinction paradigm—did not prevent spontaneous recovery of food-cup approach. Together, our studies demonstrate the critical role of conditioned orienting behavior and the CeA in updating appetitive memory during the reconsolidation window.

## Introduction

When a neutral conditioned stimulus (CS) is paired with an unconditioned stimulus (US), animals often acquire cue-directed responses, for example, approaching/orienting to a light predictive of food (Brown and Jenkins, 1968; Holland, 1977). Under certain conditions, only a subset of animals acquires cue-directed behaviors (aka sign-tracking) in addition to, or at the cost of, developing US-directed behaviors (aka goal-tracking) that ultimately lead to the obtainment of a rewarding US. Cue-directed behaviors likely reflect enhanced attentional, emotional, and/or motivational processing of the cue (Holland, 1977; Robbins and Everitt, 1996; Cardinal et al., 2002) and represent how the cues themselves can acquire incentive value (Robinson and Berridge, 2001). Several brain regions/networks, including the amygdala and dopaminergic pathways, have been implicated in cue-directed behaviors (Gallagher et al., 1990; Parkinson et al., 2000, 2002; Lee et al., 2005, 2011; Mahler and Berridge, 2009; Flagel et al., 2011). In particular, the amygdala central nucleus (CeA) and nigrostriatal circuitry are critical in mediating the conditioned orienting response (OR) directed to CSs paired with food, but are not involved in conditioned approach behavior to the food delivery site (Gallagher et al., 1990; Han et al., 1997; Lee et al., 2005; El-Amamy and Holland, 2006). These studies suggest a separate neural mechanism for cue-directed behaviors and that the nature of CS-information processing may be different in animals displaying robust conditioned cue-directed behaviors. What is not clear is how the presumably different nature of acquired CS-information influences memory extinction, retrieval and updating.

Extinction (repeated exposure to a CS that no longer predicts a US) gradually attenuates conditioned responses; however, this response attenuation is not permanent, and the conditioned responses can return in the form of renewal, reinstatement, or spontaneous recovery (Pavlov, 1927; Rescorla and Heth, 1975; Bouton and Bolles, 1979; Robbins, 1990; Bouton, 2002). Thus, extinction does not generally modify the original CS-US association, but rather creates a separate CS-noUS memory that suppresses the original memory trace (Bouton, 2004). Recently, Monfils and colleagues (Monfils et al., 2009; Schiller et al., 2010) designed an extinction paradigm for fear conditioning in rats and humans that could potentially target the original CS-US association (see also Chan et al., 2010; Clem and Huganir, 2010; Rao-Ruiz et al., 2011; Agren et al., 2012). Standard extinction trials within 6 h of a single CS exposure blocked return of conditioned fear responses. The CS exposure presumably retrieved the original CS-US memory, which was then in a labile state needing to be re-consolidated (Nader et al., 2000; Nader, 2003; Tronson and Taylor, 2007). Thus, an extinction session after the cue-induced memory retrieval possibly updated the original CS-US association to a CS-noUS association. Others have also shown that this retrieval-extinction paradigm was effective in attenuating drug-seeking behaviors (Xue et al., 2012) in both humans and rats and in suppressing conditioned reinforcement in rats (Flavell et al., 2011).

In the current study, rats were categorized as Orienters and Nonorienters based on their display of conditioned responses during the acquisition phase. Orienters displayed robust conditioned orienting/rearing to the light CS in addition to acquiring conditioned food-cup approach while Nonorienters acquired only the conditioned food-cup approach. Because both groups showed comparable goal-tracking behavior (i.e., food-cup approach), we termed them Orienters and Nonorienters (rather than sign- and goal-trackers) in order to more accurately describe their phenotypes. The first experiment examined whether the retrieval-extinction paradigm might be equally effective in blocking the return of Pavlovian appetitive responses directed to the CS (conditioned orienting/rearing response to the light) and to the US (conditioned food-cup approach). We further examined how individuals’ predilections for the cue-directed ORs might manifest in memory retrieval and extinction. In the second experiment, we investigated whether fear conditioning rather than extinction after memory retrieval could update the appetitive memory. Finally, in the third experiment, we examined the role of the CeA in appetitive memory retrieval and extinction processes given the CeA’s critical role in mediating conditioned OR.

## Materials and Methods

### Subjects

Adult male Long-Evans rats (Harlan—Experiment 1, Charles-River—Experiment 3) weighing 250–275 g upon arrival were singly housed in a reverse 14 h light/10 h dark cycle, with the lights going off at 10 am. For Experiment 2, subjects were adult male Sprague-Dawley rats (Harlan), weighing 250–275 g upon arrival and were housed in a 12 h standard light cycle with lights on at 7 am. During acclimation, water and food were available ad libitum. One week after arrival to the colony (Experiments 1 and 2) or 7–10 days post-surgery (Experiment 3), rats were put on restricted feeding to reduce weight to 90% of their free-feeding body weight; this weight was maintained throughout the study. All experiments were conducted according to the *National Institutes of Health’s Guide for the Care and Use of Laboratory Animals*, and the protocols were approved by the Institutional Animal Care and Use Committee at the University of Texas at Austin.

### Experimental Designs

#### Experiment 1: Effects of retrieval-extinction paradigm on conditioned OR and food-cup approach

In this experiment, extinction learning after memory retrieval was used to update the original appetitive memory. After animals were conditioned to light-food pairings, they received an extinction session within the reconsolidation window (i.e., a single CS exposure before standard extinction trials). Then, spontaneous recovery rate was used to measure whether the original memory was updated.

Appetitive conditioning and testing took place in eight individual conditioning chambers that had aluminum sidewalls and ceiling, with clear acrylic front and back walls (30.5 cm W × 25.4 cm D × 30.5 cm H, Coulbourn Instruments). The floor was made of stainless steel rods (0.5 cm in diameter, spaced 1.0 cm apart). The food magazine was located on the right wall of the chamber, 2.5 cm above the floor. Nose-poke entry into the magazine was detected by an infrared beam at the opening. A 2 w white light was mounted 20 cm above the food-magazine and its illumination served as a CS signaling grain pellet delivery. The left wall was concaved and had five ports with lights, which were not activated. Each chamber was enclosed in a light- and sound-attenuated box (58.4 cm × 61 cm × 45.7 cm) where the ventilation fan provided masking noise. Digital cameras were mounted within each box and images were recorded during behavioral training and testing.

Animals were first trained to eat a single grain pellet delivered to the magazine. A total of 30 pellets were delivered at a variable interval (averaging 60 s) over a 30 min session. After two pre-training sessions, all rats reliably retrieved grain pellets from the magazine. The first training session consisted of two parts. In order to habituate the unconditioned OR to light, the stimulus light was illuminated eight times, for 10 s each time, without any food pellets being delivered to the magazine. Then, during the second half of the session, eight trials of a 10 s light presentation were followed by a food pellet delivery to the magazine. For the next 3 days of conditioning, sessions consisted of 16 light—food pairings with a variable intertrial interval (ITI) averaging 120 s. Extinction occurred 24 h after the final training session. Prior to extinction, rats were pseudo-randomly divided into Retrieval and No Retrieval groups in order for each group to have similar levels of conditioned food-cup responding during acquisition. On the day of extinction, rats in the Retrieval group received one isolated CS presentation and were placed back in the home cage. After one h in the home cage, they were returned to the conditioning boxes and received 17 CS-alone presentations. Rats in the No Retrieval group underwent a typical extinction session consisting of 18 CS-alone presentations, again with a variable ITI averaging 120 s.

Both groups received a test session 24 h after extinction (Test 1), which consisted of four CS presentations, given at variable intervals (average 120 s) without delivery of a grain pellet. Three weeks after this first test session, the rats were again tested with 4 presentations of the CS alone (Test 2). In summary, training (4 days), extinction, and Test 1 were completed in 6 consecutive days. After completing Test 1, rats remained at 90% free feeding weight and were again tested 21 days after Test 1.

#### Experiment 2: Appetitive memory updating with fear conditioning after memory retrieval

Instead of using extinction learning to update the original appetitive memory, fear conditioning was used in this experiment. Thus, animals first received appetitive training, then received fear conditioning either within the appetitive memory reconsolidation window, or after appetitive memory consolidation. Subsequently, reacquisition rate of light-food pairings was used to measure the strength of the original appetitive memory.

Animals first underwent appetitive conditioning as described in Experiment 1 (Context A), except that they received an additional 16-trial training day. Forty-eight hours after the last appetitive training day, rats were fear conditioned in different conditioning chambers located in a different room (Context B). Animals were divided into Retrieval and No Retrieval groups. The same 2-w white light used during appetitive conditioning served as a CS. Rats in the Retrieval group received one CS exposure 10 min prior to fear conditioning. Rats in the No Retrieval group were placed in the conditioning context 10 min prior to the fear conditioning session, but were not exposed to a CS. Both groups of animals were held in their home cages between the CS/context exposure and fear conditioning. Then, rats were conditioned with three 10 s light CSs co-terminating with a 500 ms 0.7 mA footshock. ITI was variable, averaging 180 s. The behavior was recorded from digital cameras mounted within each chamber.

Forty-eight hours after fear conditioning, rats were placed in Context C to potentially extinguish both conditioned fear and appetitive responses to the light. Context C was created by modifying Context A chambers by inserting a smooth black floor and adding peppermint scent. Rats received 18 light-only CS presentations and conditioned appetitive (orienting and food-cup approach) and fear (freezing) responses were recorded. Seventy-two hours after extinction learning, rats were placed back in context A and received 16 light-food pairings to examine reacquisition rate.

#### Experiment 3: Role of CeA in appetitive memory updating within the reconsolidation window

Prior to behavioral training, rats first received bilateral CeA lesions. They were anesthetized with isoflurane gas (Vet Equip) and placed in a stereotaxic frame (Kopf Instruments). Two sites per hemisphere were targeted; AP −2.0/−2.4, ML 4.2, DV −8.2. Rats in the lesion group received 0.2 μL infusion (per site) of 10 mg/mL ibotenic acid dissolved in a 0.1 M phosphate buffered saline solution (PBS) (infused at 0.1 μL /min). Rats in the control group received a sham surgery consisting of either 0.2 μL infusion of PBS per site or lowering of the cannula into CeA with no infusion. Rats were allowed 7–10 days to recover before beginning food deprivation and training.

Training, retrieval-extinction, and test procedures for Experiment 3 were identical to those described in Experiment 1. However, for Experiment 3, the No Retrieval group was subdivided into a context exposure group and a no context exposure group. Animals in the context exposure group were placed in the conditioning box 1 h prior to extinction, but received no CS presentation. Animals in the no context exposure group remained in the home cage prior to extinction. As in Experiment 1, training (4 days), extinction, and Test 1 were completed in 6 consecutive days. After completing Test 1, rats remained at 90% free feeding weight and were again tested 21 days after Test 1.

Following behavioral testing, rats received an overdose of pentobarbital (86 mg/kg) and phenytoin (11 mg/kg) mix (Euthasol^®^ by Virbac Animal Health) and were perfused transcardially with 0.9% saline followed by 4% Paraformaldehyde in 0.1 M phosphate buffer (PFA). Brains were removed, post-fixed, and cryoprotected overnight in a 20% sucrose PFA. Twenty-four hours later, brains were frozen in powdered dry ice and stored at −80°C. Brains were sliced on a freezing microtome and 30 μm sections were collected. In order to verify lesion size and placement, every fourth section was mounted on slides and Nissl-stained.

### Behavioral analyses

Previous work has shown that when presented with a 10 s light CS that predicts pellet delivery, rats typically show an OR towards the light during the first 5 s (CS1) and a food-cup approach response during the last 5 s (CS2) (Holland, 1977). For all experiments, number of OR bouts were counted by a blind observer from DVD recordings of all training sessions. An OR was defined as a rearing response in which both forelimbs were lifted from the floor of the conditioning box, and did not include grooming behavior. To account for within-groups variation in baseline orienting, we report the response difference in CS1 and pre-CS (the 5 s prior to the CS). Food cup approach is reported as bouts of nose-pokes into the magazine (Experiments 1 and 3) or percentage of time spent with the nose inserted in the magazine (Experiment 2), as measured by the infrared beam. We report the difference in CS2 and pre-CS food cup responding. Freezing was scored by a blind observer and calculated as percentage of CS duration spent devoid of movement, excluding breathing and whisker twitching.

### Statistical analyses

For acquisition analyses of three experiments, orienting classification × trial repeated ANOVAs were conducted for orienting and food-cup responses. For extinction analyses, orienting classification × retrieval condition × trial repeated ANOVA was conducted. When appropriate (Experiment 1), it was followed with simple ANOVA within Orienters and Nonorienters for OR. For spontaneous recovery tests, orienting classification × retrieval condition × extinction/test days repeated ANOVA was conducted. When appropriate, it was followed up with separate ANOVAs with just Orienters or Nonorienters (Experiment 1) or a priori comparison (Experiment 3). In Experiment 2, for both fear acquisition and extinction, and appetitive reacquisition, an orienting classification × retrieval condition × trial repeated ANOVA was conducted. When appropriate, the significant interaction effects were followed up with one-way ANOVAs and then with Bonferroni tests.

## Results

### Experiment 1

#### Acquisition

**Figure 1:**
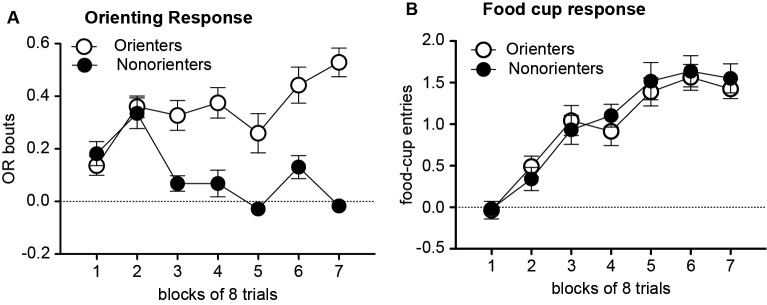
Mean (±SEM) OR **(A)** and food-cup response **(B)** during training. OR bouts were measured during the first 5 s of each CS and food-cup entries were measured during the last 5 s CS period. The values shown are elevation scores, calculated by subtracting pre-CS baseline responding from responding during the CS. Orienters, but not Nonorienters, acquired conditioned OR to the light CS, *p* < 0.0001 **(A)**. In contrast, both Orienters and Nonorienters acquired conditioned food-cup responding **(B)**.

During the conditioning sessions, in which the light cue was repeatedly paired with food, there was an overall acquisition of conditioned OR and food-cup approach behavior. However, a subset of rats did not acquire conditioned OR. Thus, based on their average number of OR bouts during the last eight trials of training, rats were divided into two groups. Rats scoring at or above the median number of OR bouts were classified as Orienters (*n* = 26), while those rats that scored below the median score were classified as Nonorienters (*n* = 22). As shown in Figure 1A, Orienters acquired conditioned OR to the light CS while Non-orienters did not show an increase in OR as training progressed. An orienting classification × trial block repeated ANOVA of OR showed a significant main effect of orienting classification, *F*(1, 42) = 46.0, *p* < 0.0001, a significant main effect of trial block, *F*(6, 252) = 9.24, *p* < 0.001, and significant interaction effects between the orienting classification and trial block, *F*(6, 252) = 9.24, *p* < 0.001. Importantly, Orienters and Nonorienters did not differ in their display of unconditioned OR. Both groups equally showed unconditioned OR at the beginning of the habituation trials and this unconditioned OR decreased over the course of the eight habituation trials: the average OR scores of the first four trials were 0.19 (Orienters) and 0.22 (Nonorienters), and the last four trials were 0.10 (Orienters) and 0.13 (Nonorienters). This was supported by a lack of main effect of orienting classification as well as orienting classification × trial interaction (*ps* > 0.05). Due to a video equipment malfunction, four rats were missing OR data from the eight habituation trials and first eight trials of training and were excluded from analysis of OR data during habituation and training. The generally low levels of conditioned OR by Orienters (Figure 1A) partly reflect the nature of OR scoring and analyses procedures. Rats typically rear once towards the light within the first 5 s but not at every trial, resulting the average score to be lower than one. In addition, even though it is not frequent, any baseline rearing during the 5 s prior to the light onset has been subtracted, resulting in negative OR scores at some trials.

In contrast to conditioned OR acquisition, both Orienters and Nonorienters showed an increase in food-cup responding as training progressed and there was no difference in acquisition rate between these two groups (Figure 1B). An orienting classification × trial block repeated ANOVA of food-cup responding showed only main effect of trial block, *F*(6, 276) = 43.3, *p* < 0.001.

#### Extinction

**Figure 2:**
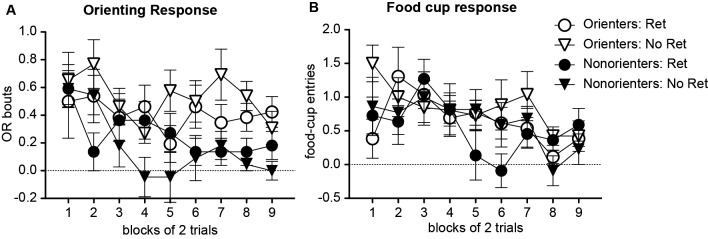
Mean (±SEM) OR **(A)** and food cup response **(B)** during extinction. Orienters and Nonorienters refer to the animals that showed robust and no conditioned orienting, respectively, during conditioning phase. Ret refers to the extinction condition, in which a single CS was presented prior to regular extinction trials while No Ret refers to the regular extinction trials without a prior CS presentation. Orienters showed more OR than Nonorienters **(A)**. There was no difference in food-cup responding among four groups, and all showed comparable extinction rates **(B)**.

For an extinction session, animals were further divided into groups that received a single CS exposure an hour prior to standard extinction trials (Retrieval group) or only standard extinction trials (No Retrieval group). Thus, there were four groups of animals: Orienters-Retrieval (*n* = 13), Orienters-No Retrieval (*n* = 13), Nonorienters-Retrieval (*n* = 11), and Nonorienters-No retrieval (*n* = 11). As expected, Orienters showed more OR than Nonorienters, but the retrieval trial did not affect extinction rates (Figure 2A). An orienting classification × retrieval conditions × extinction trials repeated ANOVA supported this observation; there was a main effect of extinction trials, *F*(8, 352) = 2.36, *p* < 0.05 and a main effect of orienting classification, *F*(1, 44) = 15.3, *p* < 0.0001, but no interaction effects among orienting classification, retrieval conditions and/or extinction trials. Even though the interaction effect of orienting classification and extinction trials was not significant (*p* = 0.17), the main extinction trial effect seemed to be driven by Nonorienters. Thus, we ran separate ANOVAs on Orienters and Nonorienters. The results show that the trial effect was only significant among Nonorienters, *F*(8, 160) = 3.43, *p* = 0.001, but not among Orienters, *F*(8, 192) = 0.98, *p* > 0.4. In terms of conditioned food cup responding (Figure 2B), all animals showed a reduction of food cup responding over the course of extinction trials, *F*(8, 352) = 4.31, *p* < 0.05, and there was no difference among the four groups, as shown by no main or interaction effects, *ps* > 0.1.

#### Test

**Figure 3:**
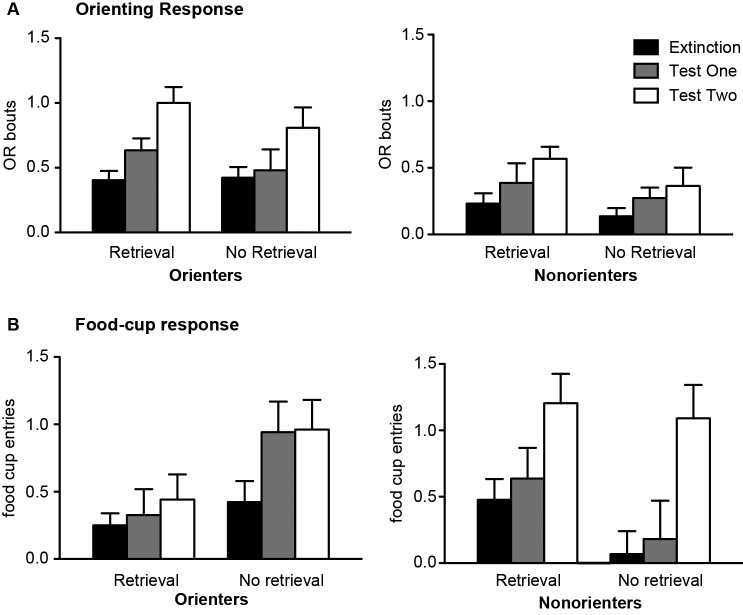
Mean (±SEM) OR **(A)** and food cup response **(B)** for Orienters (left panels) and Nonorienters (right panels). The values are responses during the last four CS alone presentations in extinction session, four CS alone presentation 24 h (test 1) and 21 days (test 2) after extinction. A single CS presentation 1 h prior to extinction trials (retrieval condition) blocked return of spontaneous food-cup response only in Orienters.

Both 24 h (Test 1) and 21 days (Test 2) after extinction, rats were tested with 4 CS exposures. In order to determine whether there was spontaneous recovery of OR and food-cup responding, the responses during the last four trials of extinction were compared to the responses during the test trials. Conditioned OR was observed in most of the animals regardless of extinction conditions (Figure 3A). As expected, Orienters generally showed higher levels of OR compared to Nonorienters. In support of this observation, an orienting classification × retrieval condition repeated ANOVA over extinction, Test 1, and Test 2 trials showed a main effect of orienting classification *F*(1, 44) = 15.0, *p* < 0.0001 and main effect of extinction-test days *F*(2, 88) = 16.5, *p* < 0.001, but no main effect of retrieval condition *F*(1, 44) = 1.92, *p* > 0.1. Furthermore, there was no interaction of orienting classification × extinction-test days, *F*(2, 88) = 1.25, *p* > 0.2, no interaction of retrieval condition × extinction-test days, *F*(2, 88) = 1.0, *p* > 0.3, and no three way interaction of orienting classification, retrieval condition and extinction-test days, *F*(2, 88) = 0.04, *p* > 0.9.

Conditioned food-cup responding was different based on orienting classification and the retrieval condition (Figure 3B). Orienters in the No Retrieval group showed similarly increased food-cup responding at both Test 1 and Test 2. By contrast, Orienters in the Retrieval group did not show much food-cup responding at either test points. Food-cup responding of Nonorienters in both Retrieval and No Retrieval groups increased during Test 2. In support of these observations, orienting classification × retrieval conditions repeated ANOVA over extinction, Test 1 and Test 2 trials showed a main effect of extinction-test days, *F*(2, 88) = 10.2, *p* < 0.0001, an interaction effect of orienting classification × extinction-test days, *F*(2, 88) = 3.16, *p* < 0.05, and an interaction effect of orienting classification × retrieval conditions, *F*(1, 44) = 9.37, *p* < 0.01. The interaction effects were further examined with follow-up analyses (i.e., retrieval condition × extinction/test days repeated ANOVA) conducted on Orienters and Nonorienters separately. Among Orienters, there was a main effect of retrieval condition, *F*(1, 24) = 6.74, *p* < 0.05, but no longer a significant main effect of test days, *F*(2, 48) = 2.53, *p* > 0.05. Among Nonorienters, there was only a main effect of test days, *F*(2, 40) = 8.82, *p* = 0.001 and no main effect of retrieval condition *F*(1, 20) = 3.14, *p* > 0.05. The results suggest that the retrieval-extinction paradigm reduced food-cup responding among Orienters but not in Nonorienters.

### Experiment 2

#### Appetitive conditioning

**Figure 4:**
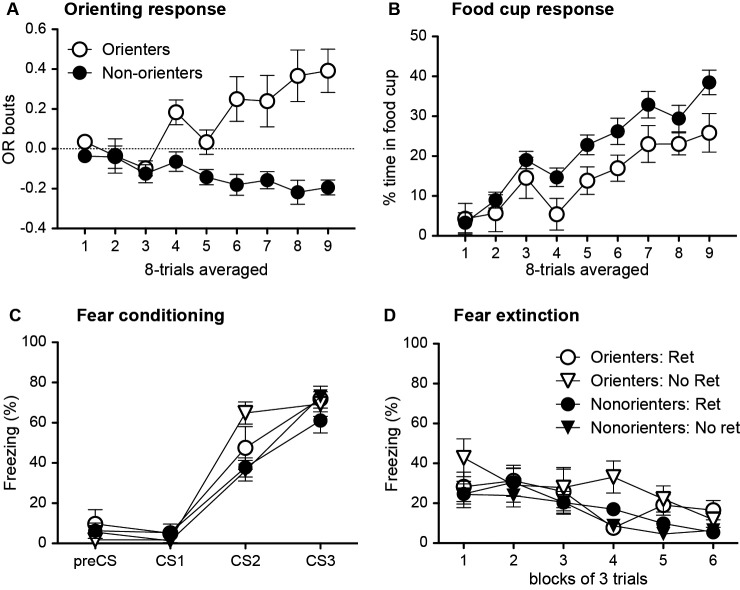
Mean (±SEM) OR **(A)** and food-cup response **(B)** during appetitive training, and freezing response during fear conditioning **(C)** and subsequent extinction trials **(D)**. Orienter and Nonorienter designations refer to those rats that developed a robust OR during appetitive training (Orienters) and those that did not (Nonorienters). Ret refers to the condition in which rats received a single CS exposure 10 min prior to fear conditioning, while No ret designates those rats were only exposed to the conditioning context prior to fear conditioning. Both Orienters and Nonorienters acquired conditioned food cup response **(B)** while only Orienters showed conditioned OR **(A)**. Both Orienters and Nonorienters achieved comparable freezing levels by the end of fear conditioning trials **(C)** and displayed similar extinction rates **(D)** regardless of retrieval condition. However, the Orienters-No Retrieval group showed slightly increased freezing levels both during acquisition and extinction trials.

During the conditioning sessions, in which the light cue was repeatedly paired with food, a subset of rats did not acquire conditioned OR (Figure 4A). Thus, based on their average number of OR bouts during the last eight trials of training, rats were divided into two groups. Rats scoring above the median number of OR bouts were classified as Orienters (*n* = 15), while those rats that scored at or below the median score were classified as Nonorienters (*n* = 31). Because a large number of rats failed to acquire the conditioned OR and displayed zero or fewer bouts of orienting, there were more Nonorienters than Orienters. An orienting classification × trial block repeated ANOVA of OR showed a significant main effect of orienting classification, *F*(1, 29) = 30.2, *p* < 0.0001, and a significant interaction effect between the orienting classification and trial block, *F*(8, 232) = 5.42, *p* < 0.0001. In contrast to the acquisition of conditioned OR, both groups acquired conditioned food-cup (Figure 4B). However, animals in the Nonorienter group showed slightly higher acquisition rate than the ones in the Orienter group. This is not unusual in that slightly higher food-cup responses have been observed at times among rats displaying attenuated OR due to brain manipulations (Gallagher et al., 1990; Han et al., 1997). An orienting classification × trial block repeated ANOVA of food-cup responding supported this observation. There was a significant main effect of trial block, *F*(8, 352) = 21.9, *p* < 0.0001, as well as a main effect of orienting classification, *F*(1, 44) = 5.65, *p* < 0.05.

#### Fear conditioning

Fear conditioning was conducted in a different context and rats were further divided into two groups in which one received a single CS exposure prior to fear conditioning (Retrieval group) while the other was only exposed to the conditioning context without CS exposure prior to fear conditioning (No Retrieval group). Then, rats in all groups received three light-footshock pairings and showed an increase in freezing to the light across three trials (Figure 4C). An orienting classification × retrieval condition × trial repeated ANOVA of percent freezing revealed significant main effects of both orienting classification, *F*(1, 42) = 6.10, *p* < 0.05, and trial, *F*(2, 84) = 155.7, *p* < 0.0001, as well as an interaction between orienting classification and trial, *F*(2, 84) = 3.67, *p* < 0.05. One-away ANOVA for each trial revealed that the groups only differed at trial 2, *F*(3, 42) = 4.65, *p* < 0.01. Follow-up Bonferroni comparisons at trial 2 showed that Orienters in No Retrieval group, but not in Retrieval group, displayed significantly higher freezing compared to Nonorienters in both Retrieval (*p* = 0.01) and No Retrieval (*p* = 0.01) groups. However, all four groups of animals displayed comparable freezing by the end of fear conditioning as shown by non significant effect at the third trial, *F*(3, 42) = 1.07, *p* > 0.1.

#### Extinction

In a context that was different from the ones used for either appetitive and fear conditioning, an extinction session of 18 light-alone trials was given to assess both appetitive and fear responses as measured by conditioned OR, food-cup approach, and freezing. If fear conditioning after CS retrieval updated the original appetitive memory, then higher freezing levels and lower appetitive behaviors should be seen in the retrieval group, particularly among Orienters. We predicted that the rats in the no retrieval group would predominantly display fear responses initially, but might display appetitive responses as fear responses extinguished. Thus, we hypothesized that differences in fear and appetitive responses would be observed at the beginning and the end of extinction trials, respectively.

Contrary to our prediction, the retrieval condition neither yielded higher fear responses nor lower appetitive behaviors compared to no retrieval condition. Overall, all rats showed comparable freezing levels and extinction rate as shown by the main effect of trial block, *F*(5, 195) = 10.9, *p* < 0.0001 without any interaction effects (Figure 4D). Interestingly, there was a main effect of orienting classification, *F*(1, 39) = 4.24, *p* < 0.05, which is likely to be driven by higher freezing levels seen in the Orienters-No Ret group. One-way ANOVA for each trial revealed that the groups only differed at trial blocks 4 and 5, *F*(3, 42) = 6.0, *p* < 0.01 and *F*(3, 42) = 3.97, *p* < 0.05, respectively. A post-hoc Bonferroni revealed that the Orienter-No Ret group froze significantly more than Orienter-Ret and Nonorienter-No Ret groups at trial block 4 (*ps* < 0.01) and from the Nonorienter-No Ret group at trial block 5 (*p* < 0.05). In contrast to our prediction, appetitive responses did not re-emerge as freezing extinguished in any of the groups. Rats displayed very few appetitive behaviors throughout the session; the overall average of OR bout was −0.05 and percent food-cup response was 1.63.

#### Appetitive retraining

**Figure 5:**
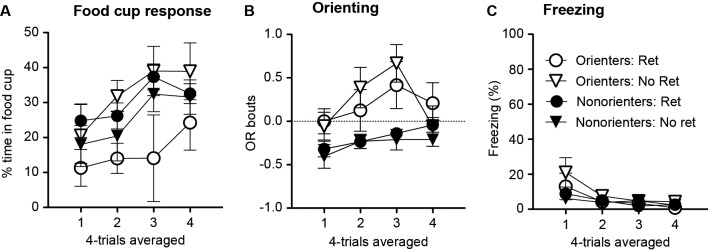
Mean (±SEM) food cup response **(A)**, OR **(B)** and freezing **(C)** during the appetitive reacquisition phase. Orienter and Nonorienter designations refer to those rats that developed a robust OR during the original appetitive training (Orienters) and those that did not (Nonorienters). Ret refers to the condition in which rats received a single CS exposure 10 min prior to fear conditioning while No ret designates those rats that did not (context exposure only). Only Orienters in the retrieval condition showed retarded reacquisition of conditioned food cup response **(A)**, but intact reacquisition of conditioned OR **(B)** and no difference in the minimal levels of freezing **(C)**.

To test for savings of the original appetitive memory, rats were retrained in the original context with 16 light-food pairings. If fear conditioning after CS retrieval updated the original appetitive memory, then slower reacquisition of appetitive behaviors should be seen in the retrieval group, particularly among Orienters. Given that extinction after CS retrieval blocked spontaneous recovery only for Orienters in Experiment 1, we predicted that fear conditioning after CS retrieval would be more effective in updating appetitive memory with fear memory only for Orienters. In support of our hypothesis, the retrieval condition as well as orienting classification played an important role in reacquisition of conditioned food-cup approach (Figure 5A). An orienting classification × retrieval condition × trial repeated ANOVA revealed that there was an overall reacquisition of food-cup behavior among all four groups, *F*(3, 126) = 6.11, *p* = 0.001. However, the Orienters in the Retrieval condition showed a retarded reacquisition rate. This observation was supported by the interaction effect of orienting classification and retrieval condition, *F*(1, 42) = 6.23, *p* < 0.05. A follow-up one way repeated ANOVA among Orienters revealed a main effect of retrieval condition, *F*(1, 13) = 4.71, *p* < 0.05 but not among Nonorienters, *F*(1, 29) = 1.0, *p* > 0.3. When considering OR, the retrieval condition did not influence reacquisition rate. Overall, the Orienters displayed reacquisition of conditioned OR while the Nonorienters did not (Figure 5B). An orienting classification × retrieval condition × trial block repeated ANOVA confirmed this observation. There was a significant main effect of trial block, *F*(3, 126) = 6.14, *p* < 0.001, a main effect of orienting classification, *F*(1, 42) = 21.7, *p* < 0.0001, and an interaction effect of trial block and orienting classification, *F*(3, 126) = 3.12, *p* < 0.05. However, there was no interaction effect of orienting classification and retrieval condition, *F*(1, 42) = 0.48, *p* = 0.5.

As expected, minimal fear responses were displayed, but the freezing levels were slightly higher at the beginning of the trials as shown by the main effect of session, *F*(3, 126) = 15.5, *p* < 0.001 (Figure 5C) . This difference was mainly driven by the Orienters as shown by the interaction effect of orienting classification and session block, *F*(3, 126) = 4.0, *p* < 0.05. In particular, the Orienter-No Ret group showed slightly higher freezing levels at the beginning of reacquisition session. Post-hoc Bonferroni tests revealed that the Orienter-No Ret group was significant different from the two Nonorienter groups at the first trial block, *ps* < 0.05. Importantly, the Orienters in the retrieval condition did not show any differences in the minimal display of conditioned freezing compared to the other three groups, suggesting that the retarded reacquisition of conditioned food-cup response was not simply due to higher freezing response.

### Experiment 3

#### Histology

**Figure 6:**
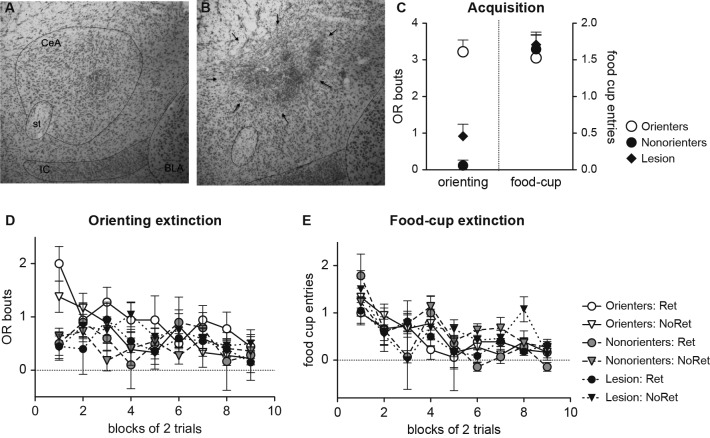
Representative photomicrographs of the amygdala region from the animals with sham lesion **(A)** and ibotenic acid lesion **(B)**. Central amygdala (CeA), stria terminalis (ST), intercalated nucleus (IC), and BLA are highlighted. Average lesion size was 65% CeA damage, and rats with significant BLA damage were excluded. **(C)** Mean (±SEM) OR and food cup response during the last eight trials of training for Orienters, Nonorienters, and Lesion rats. Animals with CeA lesions showed minimal conditioned OR, but still showed intact conditioned food-cup response. **(D and E)** Mean (±SEM) OR and food cup response during extinction. Orienters showed more OR than Nonorienters and CeA Lesioned rats at the beginning but at the end. There was no difference in food-cup responding among six groups, and all showed comparable extinction rates.

Twenty-four lesions were deemed acceptable. Lesions were rej­ected (*n* = 10) if there was less than 30% damage to the medial CeA of either hemisphere or if there was extensive damage to surrounding areas such as the basolateral nucleus (BLA) of the amygdala. Average bilateral lesion size was 65% damage of the entire CeA. Figures 6A, B show pictures of intact and lesioned CeA.

#### Acquisition

Rats with the CeA lesions were not expected to acquire conditioned OR. Thus, only rats in the sham surgery group were divided into Orienters and Nonorienters. This division provided three groups for analysis of training data: Lesion (*n* = 24), Orienter (*n* = 18), and Nonorienter (*n* = 18). As expected, Nonorienters as well as rats with good bilateral CeA lesions did not acquire conditioned OR. A group × trial block repeated ANOVA revealed a significant main effect of trial block, *F*(6, 342) = 2.43, *p* < 0.05, but also a significant group × trial block interaction, *F*(12, 342) = 5.05, *p* < 0.001. As seen in Figure 6C, by the end of training Orienters displayed significantly higher conditioned OR when compared to Lesion rats and Nonorienters. A one-way ANOVA on the mean OR scores of the last eight trials showed a main effect of groups, *F*(2, 57 = 27.8, *p* < 0.001, and a post-hoc Bonferroni test revealed that OR scores of Orienters were significantly higher from the ones of Nonorienters (*p* < 0.001) and Lesion rats (*p* < 0.001). As expected, there was no difference between Nonorienters and Lesion rats (*p* > 0.3).

Regardless of the lesion/orienting classifications, all animals acquired the conditioned food-cup response as training progressed and no differences in acquisition rates existed among these three groups. By the end of training, all reached the same levels of conditioned food-cup approach (Figure 6C). A group × trial block repeated ANOVA showed only a main effect of trial block, *F*(6, 324) = 29.78, *p* < 0.001. There was neither a main effect of lesion/orienting classifications, *F*(2, 57) = 0.01, *p* = 0.99 nor an interaction effect of trial block by lesion/orienting classification, *F*(12, 342) = 1.31, *p* > 0.2.

#### Extinction

At the end of training, Lesion rats, Orienters, and Nonorienters were further divided into the Retrieval and No Retrieval groups. Within the No Retrieval group, half of the rats were exposed to the context without the light CS while the others remained in their home cages. A lesion/orienting classification × retrieval condition (retrieval, context exposure, no context exposure) repeated ANOVA on food-cup response revealed only a main effect of extinction trials, *F*(17, 782) = 3.03, *p* < 0.001. Even though there was no main effect of retrieval condition, we did further analyses comparing just the context and no context exposure (i.e., orienting/lesion classification × context exposure repeated ANOVA with extinction trials) to make sure there was still no difference when these two factors were directly compared. There was neither a main effect of context exposure, *F*(1, 27) = 0.67, *p* > 0.4, nor an interaction effect of context exposure by orienting/lesion classification, *F*(2, 27) = 2.39, *p* > 0.1. Therefore, the context and no context exposure groups were collapsed as the No Retrieval group. There were thus six groups; Lesion-Retrieval (*n* = 11), Lesion-No Retrieval (*n* = 13), Orienters-Retrieval (*n* = 9), Orienters-No Retrieval (*n* = 9), Nonorienters-Retrieval (*n* = 7), Nonorienters-No Retrieval (*n* = 11).

As expected, Orienters displayed more OR responses at the beginning of the extinction session compared to Nonorienters or Lesion rats (Figure 6D). However, the overall OR decreased throughout extinction and groups were not significantly different at the end of the session. A lesion/orienting classification × retrieval condition × trial repeated ANOVA confirmed a significant main effect of trial, *F*(17, 918) = 2.23, *p* < 0.05, as well as a lesion/orienting classification × trial interaction, *F*(34, 918) = 1.62, *p* < 0.05. One-way ANOVA on the mean OR scores of the first two trials showed a main effect of groups, *F*(2, 57) = 11.2, *p* < 0.001, and a post-hoc Bonferroni test revealed that OR scores of Orienters were significantly higher from the ones of Nonorienters (*p* = 0.001) and Lesion rats (*p* < 0.001). When the last two trials of OR scores were analyzed, there was no main effect of lesion/orienting classification, *F*(2, 57) = 0.29, *p* > 0.7. In contrast to OR responding, the food-cup approach did not differ among Orienters, Nonorienters, and Lesion rats (Figure 6E). A lesion/orienting classification × retrieval condition × trial repeated measures ANOVA revealed only a significant main effect of trial, *F*(17, 918) = 3.27, *p* < 0.001.

#### Test

Four rats (2 in the Orienter-No retrieval group, 1 in the Nonorienter-Retrieval group, and 1 in the Lesion-Retrieval group) did not receive light-CS exposures during Test 1. They were placed in the context, but a computer malfunction resulted in no light exposures. Because their behaviors did not differ from their cohorts in Test 2, their Test 2 data were included. Thus, we ran orienting classification × retrieval conditions repeated ANOVA over extinction and Test 2 only. Including Test 1 as a repeated factor by eliminating those 4 rats did not change the results.

**Figure 7:**
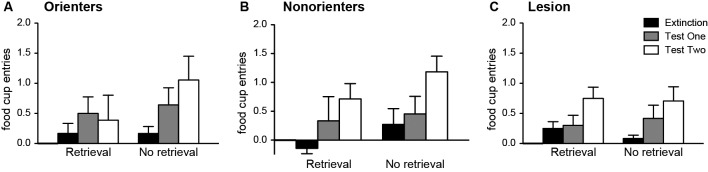
**Mean (±SEM) food-cup responding during extinction and tests both 24-h (Test 1) and 21 days (Test 2) after extinction**. The values are responses during the last two CS alone presentations of the extinction session, and the first two CS alone presentations during Test 1 and Test 2. Orienters in the retrieval condition are the only animals not showing spontaneous recovery of conditioned food-cup response.

Conditioned OR was observed in most of the animals regardless of retrieval condition or orienting/lesion classifications. There was only a main effect of extinction/test days, *F*(1, 54) = 16.14, *p* < 0.001 (see Table 1 for the OR data). Similar results were found with the food-cup responses. There was only a main effect of extinction/test days, *F*(1, 54) = 21.7, *p* < 0.001 (Figure 7). Even though there were no significant interaction effects, we conducted a priori planned comparisons to confirm that the retrieval-extinction paradigm was still effective at keeping the food-cup response low for Orienters when tested 3 weeks after extinction. Paired *t*-tests between extinction and Test 2 for the Retrieval condition in each orienting/lesion classified groups confirmed no significant effect among Orienters, *t*(8) = 0.61, *p* > 0.5, but significant effects among Nonorienters, *t*(6) = 3.29, *p* = 0.0167, and Lesion rats, *t*(10) = 2.95, *p* = 0.014 after correcting for multiple comparisons (significant *p* value at 0.0167).

**Table 1:** **Mean (±SEM) orienting response during the last 2 trials of extinction and the first two trials of test done at 24-h (Test 1) and 21-days (Test 2) after extinction**.

		**Extinction**	**Test 1**	**Test 2**
**Orienters**	Ret	0.44 (0.23)	1.56 (0.30)	1.17 (0.30)
		No Ret	0.22 (0.12)	1.14 (0.43)	0.89 (0.25)
**Nonorienters**	Ret	0.33 (0.56)	1.00 (0.39)	1.07 (0.43)
		No Ret	0.36 (0.19)	0.95 (0.32)	1.45 (0.18)
**Lesion**	Ret	0.18 (0.21)	0.90 (0.24)	0.85 (0.22)
		No Ret	0.62 (0.16)	1.04 (0.18)	0.63 (0.25)

## Discussion

The current studies highlight the role of conditioned OR in cue processing, specifically in cue-associated memory retrieval and updating. Experiment 1 showed that extinction within the reconsolidation window was effective at persistently reducing conditioned food-cup approach only in those rats that showed robust conditioned OR during the acquisition phase. In addition, results from Experiment 2 suggest that fear conditioning introduced during an appetitive memory reconsolidation window altered the original CS-associated appetitive memory—Orienters in the retrieval group showed slower reacquisition of conditioned food-cup behavior when tested for savings of appetitive memory. Together these results suggest that the differences in the display of conditioned OR reflect fundamental differences in stimulus encoding, memory retrieval and updating. Finally, Experiment 3 suggests that the CeA, known to be necessary for the acquisition of conditioned OR, is critical for the retrieval-extinction paradigm to effectively block return of conditioned food-cup behavior.

### Robust effects of the retrieval-extinction paradigm in diverse procedures

It should be noted that the attenuation of conditioned food-cup response following the retrieval-extinction paradigm was replicated in Experiments 1 and 3 despite several major differences between the original Monfils et al. (2009) work and the current study. The differences included valence of the US (shock vs. food pellet), modality of the CS (tone vs. light), number of CS-US pairings (3 vs. 56), rat strain (Sprague-Dawley vs. Long-Evans), and circadian rhythm (testing in light vs. dark cycle). Indeed, within the current studies, differences existed in rat strain (Long-Evans in Experiments 1 and 3 vs. Sprague-Dawley in Experiment 2), light cycle (dark in Experiment 1 and 3 vs. light in Experiment 2), and number of appetitive CS-US pairings (56 in Experiment 1 and 3 vs. 72 in Experiment 2). Furthermore, in Experiment 2, fear conditioning rather than extinction during the reconsolidation window was used and was still effective in updating a previously acquired appetitive memory. As was the case in Monfils et al. (2009), the current study also showed that the retrieval-extinction paradigm relied on exposure to the specific CS and not on general exposure to the context. The context exposure effect was directly tested in Experiment 3 among animals in the No Retrieval group; one subgroup was exposed to the context without CS presentation while the other group remained in the home cage. Equivalent spontaneous recovery was observed in both groups. Thus, the current study suggests that the retrieval-extinction paradigm can be effective in updating appetitive memory. In fact, other recent studies have reported that the retrieval-extinction paradigm was effective in a variety of appetitive settings. For example, extinction after drug-associated cue presentation prevented drug-seeking behaviors in rats and drug craving in humans (Xue et al., 2012). In another study, rats did not acquire conditioned reinforcement with a food-associated light cue that was subjected to the retrieval-extinction paradigm (Flavell et al., 2011). However, unlike earlier findings, our results showed that the retrieval-extinction paradigm worked only in a subset of animals (Orienters). Similarly, the effectiveness of fear conditioning within the reconsolidation window in Experiment 2 was also dependent upon propensity of OR. Moreover, unlike conditioned food-cup approach behavior, conditioned OR was not affected by the retrieval-extinction/new learning paradigm in which conditioned OR was still seen during the tests (in Experiments 1 and 3) and reacquisition (in Experiment 2) among Orienters.

### Specific effects of the retrieval-extinction paradigm on food-cup response

Although both OR and food-cup approach behavior are reflective of CS-US associative strength, conditioned OR is thought to reflect attentional processing in particular (Holland, 1977; Holland and Gallagher, 1999). In support, various studies have shown independent neural processing of these two conditioned responses. Conditioned OR, but not conditioned food-cup response, relies on the CeA-nigral dopamine system (Han et al., 1997; Lee et al., 2005; El-Amamy and Holland, 2006), which has also been implicated in several behavioral tasks designed to measure attentional processing (Lee et al., 2006, 2007, 2008, 2009). Interestingly, the CeA is only required during the acquisition of conditioned OR and is unnecessary for the expression of fully acquired conditioned OR (McDannald et al., 2004). In contrast, the nigro-dorsolateral striatal circuitry is needed to express conditioned OR (Han et al., 1997; El-Amamy and Holland, 2006), suggesting a habit-like process of fully conditioned OR. Thus, extinction during the reconsolidation window may not target fully conditioned OR that relies on the dorsolateral striatum for expression. The neural circuitry underlying the conditioned food-cup response is unknown; however, the BLA, but not the CeA, is known to play an important role in encoding and representing reinforcement value of the CS (Hatfield et al., 1996). In particular, the BLA and its connections with the orbitofrontal cortex are important for updating the current value of a specific CS (Gallagher et al., 1999; Schoenbaum et al., 1999, 2003a,b). Thus, different neural circuitries contribute to different processes engaged in appetitive conditioning (Holland and Gallagher, 1999). The retrieval-extinction and retrieval-novel training paradigms, which aim to update the original CS-US association to a CS-no US and CS-new US association, respectively, might be more effective at targeting the neural process for encoding and updating CS value rather than the process important for regulating attention to CS. Interestingly, in Xue et al. (2012), the retrieval-extinction paradigm influenced protein kinase Mζ expression in the BLA, but not in the CeA.

It should be pointed out that both Nonorienters and rats with CeA lesions showed ORs comparable to Orienters during the test days (see Table 1). Both at the end of acquisition phase (Figure 6C) and at the beginning of extinction session (Figure 6D), Nonorienters and CeA lesioned rats showed significantly fewer ORs as compared to Orienters, as expected. However, during the habituation period when the light CS is presented without food, all three groups of rats displayed comparable unconditioned ORs: overall OR counts over eight trials were 2.4 (Orienters), 2.5 (Nonorienters), and 2.2 (CeA lesion). In accord, previous work (and the current study) has repeatedly shown neural and behavioral dissociations between unconditioned and conditioned orienting (Gallagher et al., 1990; Lee et al., 2005, 2011). Thus, one possibility is that the return of orienting seen during the tests might partly reflect unconditioned orienting. Our interpretation of this finding is limited in the current form and further investigation is needed.

### Individual variations in the display of conditioned orienting and memory updating

Even though the retrieval-extinction/new learning did not influence conditioned OR, the effectiveness of this paradigm at persistently reducing conditioned food-cup behavior was influenced by the animals’ propensity to display conditioned OR. Others have shown individual differences in the display of cue-approach behavior, also termed sign-tracking (see Flagel et al., 2009 for review) and reported behavioral and physiological differences seen in sign-trackers. For example, different monoamine activities in mesolimbic system (Tomie et al., 2000; Flagel et al., 2007, 2010, 2011), elevated corticosterone levels (Tomie et al., 2000), enhanced cocaine-induced psychomotor sensitization (Flagel et al., 2008), and high impulsivity (Tomie et al., 1998; but see Lovic et al., 2011) have been reported in sign-trackers. Our unpublished work also suggests that Orienters make more impulsive decisions and show enhanced 50-kHz ultrasonic vocalization in response to amphetamine. While some specific circuitries remain unknown, dopamine neurotransmission appears to be involved in all forms of sign-tracking behaviors. In particular, Flagel et al. (2011) showed an interaction of dopamine and cue-approach behavior: dopamine release in the nucleus accumbens following the CS was associated with animals showing prepotent sign-tracking behavior and intact dopamine function was necessary for the acquisition of sign-tracking. These data suggest that animals with a natural tendency to develop cue-approach behavior encode and process stimulus information differently from animals that do not show robust cue-approach behavior.

In the current studies, presumably enhanced attention to the CS (as measured by heightened conditioned OR) may allow for complete retrieval of the original CS-US memory, subsequently making that memory more apt for updating during extinction or new learning. Given that the CeA-nigral dopamine circuitry is essential for the acquisition of conditioned OR (Lee et al., 2005; El-Amamy and Holland, 2006), rats that show a natural tendency to develop a prepotent conditioned OR may have enhanced CeA-nigral dopamine function. Under normal extinction trials (or new learning), the CeA-nigral circuitry’s role may not be as important, as typical extinction (or new learning) most likely does not rely upon retrieval of a previously acquired CS-US memory. However, enhanced CeA-nigral dopamine function may aid extinction (or new learning) during reconsolidation by enhancing cue-induced retrieval of CS-US associative memory and updating it to a CS-no US memory or, in the case of novel training following retrieval, a CS-new US memory. This view is supported by findings from Experiment 3, as rats with CeA lesion showed food-cup responding 3 weeks following retrieval-extinction, an indication that they were unable to permanently update the value of the CS. A future study will be needed to address whether the intact CeA function is necessary at the time of appetitive acquisition and/or during memory retrieval-extinction.

We also observed a trend in differences of food-cup approach between Orienters and Nonorienters when they were tested a day after extinction: Orienters showed substantial conditioned food-cup approach, which was not evident among Nonorienters (Test 1 data of No Retrieval group in Figure 1B). The observed conditioned food-cup approach in Orienters-No Retrieval group during Test 1 was only marginally significant compared to its own food-cup behavior seen at the end of extinction (*p* = 0.094), but was significantly different (without correcting for multiple comparisons) from the food-cup behavior seen in Nonorienters-No Retrieval group at Test 1 ( *p* = 0.047). However, this observation was not replicated in Experiment 3, questioning the consistency of this particular phenomenon observed between Orienters and Nonorienters. Nonetheless, the retrieval-extinction paradigm was effective at keeping the conditioned food-cup approach low at both Test 1 and 2 for Orienters. More work is needed to examine the potential orienting phenotypic differences in maintenance of extinguished food-cup behavior, which can have implications in the interpretations of how the retrieval-extinction paradigm reduces food-cup behavior persistently.

### Conditioned orienting and fear learning

In Experiment 2, Orienters in No Retrieval group displayed higher conditioned freezing levels generally. They showed rapid acquisition rate of fear conditioning, better long-term memory (seen in the first block of fear extinction), and reduced extinction learning. It is interesting that the enhanced conditioned freezing is not seen among Orienters that were fear conditioned after memory retrieval (i.e., receiving a single presentation of the CS previously paired with food). Because rats in the Retrieval group were exposed to an additional presentation of the light, we cannot rule out the possibility that exposure to a single unreinforced CS itself (independent of the retrieval effect) had an impact on subsequent fear conditioning and memory updating. Interestingly, the enhanced freezing in No Retrieval group compared to Retrieval group was not observed among Nonorienters. What should be noted though is that despite slightly lower conditioned fear in Retrieval group compared to No Retrieval group among Orienters, fear learning in the Retrieval group had a more profound effect on the original appetitive memory. Appetitive reacquisition was significantly lower in Orienters-Retrieval group, suggesting successful updating of CS associative memory in this group.

Rats in the No Retrieval condition that received light-food pairings first and then light-footshock pairings are likely to form two separate appetitive and aversive memory for the same light CS. Perhaps, Orienters with already enhanced attention to the light CS are better at forming parallel associations for the same CS. A recent study also reported that sign-tracking animals showed enhanced conditioned fear to a discrete tone cue (Morrow et al., 2011). Interestingly, the same study showed that sign-tracking animals were worse than goal-tracking animals in contextual fear conditioning. Unlike our study, in which the same light CS was used for appetitive and aversive conditioning, Morrow et al. (2011) used two different CSs for appetitive and aversive conditionings (i.e., insertion of a lever paired with food and tone/context paired with footshock). However, in our other work published in the same issue (Olshavsky et al., 2013), we saw no difference in conditioned freezing between Orienters and Nonorienters when a different tone CS was used for fear conditioning with 0.7 mA footshock. Interestingly, when 1.0 mA footshock was used in the same study (Olshavsky et al., 2013), Nonorienters displayed more post-shock freezing. The discrepant results could partially be due to procedural differences and deserve further investigation. For example, our work used three presentations of 500 ms 0.7 mA (or 1 mA) footshock while the work by Morrow et al. (2011) used five presentations of 2 s 1.0 mA footshock. It is also plausible that the two forms of sign-tracking behaviors, conditioned orienting and lever-approach, rely on different neural mechanisms (as discussed earlier) and therefore reflect different phenotypes.

### Mechanisms of the retrieval-extinction paradigm

Even though the current study is limited in providing mechanistic explanation, it contributes to our understanding of the retrieval-extinction paradigm on memory maintenance and opens the door for many follow-up experiments to be conducted, in the appetitive as well as fear fields. One possible explanation of the current results is that the retrieval-extinction manipulation works via memory updating mechanism. In Monfils’ 2009 work, GluR1 phosphorylation in the lateral nucleus of the amygdala was increased following a single CS presentation, but returned to baseline levels after the administration of a second CS 1 h, but not 3 min, after the first. Other studies (Clem and Huganir, 2010; Rao-Ruiz et al., 2011) also provided evidence consistent with the results and mechanistic explanation Monfils provided in 2009 and 2010 in the follow up study in humans (Schiller et al., 2010).

Recently, Baker et al. (2013) showed that a single CS presentation either before or after a standard extinction session (i.e., retrieval + extinction or extinction + retrieval) essentially produced the same effect. They suggested that these two manipulations were driven by the same mechanism; that is some form of facilitation and/or strengthening of extinction would be occurring due to the spacing of the stimuli. We believe that the retrieval + extinction and extinction + retrieval, though they yield similar behavioral outcomes, are likely to operate through different mechanisms—the retrieval-extinction is due to an updating during reconsolidation, and the extinction + retrieval is due to extinction facilitation/strengthening. The study by Baker et al. (2013) does not allow for a distinction in mechanisms, since they only tested behavior (freezing). Published data from our lab as well as others generally point to the latter interpretation of memory updating (Monfils et al., 2009; Clem and Huganir, 2010; Rao-Ruiz et al., 2011). Nevertheless, Baker et al.’s approach is an interesting one and contributes to the field by introducing potential factors that can influence extinction and memory updating. For example, the Baker et al. study study found the retrieval-extinction effect in young adolescent rats while their earlier study did not find the retrieval-extinction effect in adult rats (Chan et al., 2010). Our current study tried to address whether the retrieval + extinction effect on fear conditioning was generalizable to another form of learning, but also aimed to understand some of the boundary conditions that may be contributing to the variability in reported effects from various groups.

### Implications

Work investigating how CSs elicit and maintain certain conditioned responses is important in delineating the psychological processes and neural mechanisms that contribute to drug addiction. Accumulating evidence suggests an important role of associative learning processes in drug addiction, in which the environmental cues become associated with reinforcing effects of a drug and later induce a vulnerable state of drug craving and elicit drug-seeking behaviors (Everitt et al., 1999; Weiss et al., 2001; Wise, 2004; Hyman et al., 2006; Robbins et al., 2008; Robinson and Berridge, 2008; Belin et al., 2009). Thus, weakening or undoing the cue-drug association can potentially prevent drug relapse (Taylor et al., 2009). In fact, Xue et al. (2012) showed that the retrieval-extinction paradigm was effective in reducing drug craving and relapse. However, they reported that the drug seeking behavior was only reduced, and not completely blocked, in some cases. Our study suggests that individual differences in cue-directed behavior may affect memory retrieval and updating of CS-associated memory differently. Thus, treatments for drug addiction based on the retrieval-extinction paradigm might work more effectively in a subset of populations. Further studies will be necessary to understand if individual differences in processing discrete CS-associated memory can be used effectively to target drug-associated memory.

## Author contributions

Megan E. Olshavsky designed, conducted and wrote the work. Bryan J. Song designed and conducted the work. Daniel J. Powell and Carolyn E. Jones helped with the experiments and data analyses. Marie-H. Monfils and Hongjoo J. Lee designed and wrote the work.

## Conflict of interest statement

The authors declare that the research was conducted in the absence of any commercial or financial relationships that could be construed as a potential conflict of interest.
